# Tribological Properties of Glass Bead-Filled Polyamide 12 Composite Manufactured by Selective Laser Sintering

**DOI:** 10.3390/polym15051268

**Published:** 2023-03-02

**Authors:** Abdelrasoul Gadelmoula, Saleh Ahmed Aldahash

**Affiliations:** 1 Department of Mechanical and Industrial Engineering, College of Engineering, Majmaah University, Al-Majmaah 11952, Saudi Arabia; 2 Department of Mechanical Design and Production Engineering, Faculty of Engineering, Assiut University, Assiut 71515, Egypt

**Keywords:** selective laser sintering, tribological properties, PA 3200 GF, build orientation, friction-induced noise

## Abstract

To enhance the properties of polyamide 12 (PA12/Nylon 12) manufactured by the selective laser sintering (SLS) process, micron-sized glass beads are used as a filler, and the resulting composite is known as glass bead-filled PA12 (PA 3200 GF). Despite PA 3200 GF basically being a tribological-grade powder, very little has been reported on the tribological properties of laser-sintered objects based on this powder. As the properties of SLS objects are orientation-dependent, this study is devoted to investigating the friction and wear characteristics of the PA 3200 GF composite sliding against the steel disc in the dry-sliding mode. The test specimens were aligned in the SLS build chamber along five different orientations (X-axis, Y-axis, Z-axis, XY-plane, and YZ-plane). Additionally, the interface temperature and the friction-induced noise were measured. The pin-shaped specimens were examined using a pin-on-disc tribo-tester for 45 min to investigate the steady-state tribological characteristics of the composite material. The results revealed that the orientation of build layers relative to the sliding plane was a ruling parameter that determined the dominant wear pattern and the wear rate. Accordingly, where build layers were parallel or inclined to the sliding plane, abrasive wear predominated, and wear rate became 48% higher than that of specimens with perpendicular build layers, for which adhesive wear predominated. Interestingly, a noticeable synchronous variation of adhesion and friction-induced noise was observed. Taken together, the results from this study can efficiently serve the goals of fabricating SLS-functional parts with customized tribological properties.

## 1. Introduction

The selective laser sintering (SLS) process is an additive manufacturing (AM) technique which allows the fabrication of polymer-based complex 3D objects. SLS is a layer-by-layer manufacturing technique in which the 3D CAD file of the object is sliced into discrete successive layers of predetermined thickness. During fabrication, the polymer powder is heated to a temperature close to its glass transition temperature (in case of amorphous polymers) or close to the melting temperature (in case of semi-crystalline polymers), and a fresh layer of powder is spread over the build area. Then, the laser beam scans a selected area corresponding to the geometry of the sliced CAD model. The supplied laser energy causes the powder particles to fuse together, forming the first layer of the object. After that, the build platform is lowered by a layer thickness, and a new, fresh powder layer is spread over the build area. The process is repeated until the whole object is fabricated as consolidated successive layers [1]. Finally, the printed object is extracted from the build chamber and cleaned to remove any sticking raw powder particles. Unlike other 3D printing techniques, sintered parts are self-supported, since the unfused powder acts as a support for the fabricated object. Additionally, a binder to the build base is not needed in SLS, which makes it suitable for the fabrication of biomedical objects. Due to the very fast printing, high-dimensional accuracy, low-manufacturing cost compared with similar technologies, and high material reuse rate, SLS has evolved from a rapid prototyping technique to the point that it is being used for the manufacturing of functional parts with low-to-moderate production volumes [2]. Indeed, due attention must be paid when setting the fabrication parameters to avoid unacceptable fabrication faults, such as edge warpage, excessive heating, porous microstructure, and rough surface. Fabrication parameters can be classified into two categories: (1) parameters related to powder material, such as the powder bed temperature, laser power, scan spacing, scanning speed, and layer thickness and (2) parameters related to the fabrication system, such as build orientation, hatching pattern, and post-processing [3,4,5].

Several thermoplastic powders are now available for SLS; however, polyamide 12 (PA12) is the most commonly used powder for SLS, due to its favorable sintering properties, which include good powder flowability, low melt viscosity, and a wide range between the melting and crystallization temperatures [6]. Consequently, laser-sintered PA12 functional parts are used in different applications, including polymer/metal tribo-contacts, despite its relatively high coefficient of friction [7]. Therefore, fillers (particulates or fibers) have been introduced to improve the mechanical and/or tribological properties of laser-sintered PA12 [8,9]; among such fillers are the micron-sized glass beads (soda lime glass). The resulting powder is being used commercially in SLS as PA 3200 GF or PA12-GB, with 30% glass bead content. Because of its improved stiffness and thermal stability, laser-sintered PA 3200 GF finds its applications in bearing housings, enclosures, support structures, and thermally loaded parts. Additionally, because of its marked abrasion resistance, PA 3200 GF composite can be used to reduce friction and wear rates in polymer/metal tribo-contacts.

Despite PA 3200 GF being mainly a tribological-grade powder, most research on glass-bead-filled PA12 focus on evaluating the mechanical properties of laser-sintered objects [10,11,12,13,14], and less effort has been directed towards studying its tribological properties [15,16]. Furthermore, as the mechanical properties of laser-sintered PA12 are orientation-dependent [17,18,19,20,21,22], as well as its tribological properties [23,24], effects of build orientation on the tribological properties of laser-sintered PA 3200 GF composite should be further assessed. Therefore, the present work aims to investigate the effect of part build orientation on the tribological properties (manifested by the coefficient of friction and wear rate) of a laser-sintered, glass bead-filled PA12 (PA 3200 GF) composite sliding against the steel countersurface in dry-sliding mode. Additionally, frictional heating, as well as the friction-induced noise level, are measured. The results are expected to declare insights about the appropriate build orientations for the optimum tribo-contact behavior of the PA 3200 GF composite, which can help in tailoring SLS objects with customized tribological properties.

## 2. Experimental Methods

### 2.1. SLS of Test Specimens

**Figure 1:**
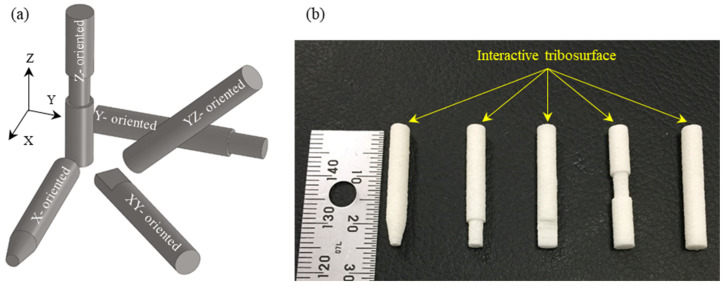
(**a**) Schematics of specimens’ orientation in the SLS build chamber; (**b**) fabricated SLS specimens, PA3200GF.

**Figure 2:**
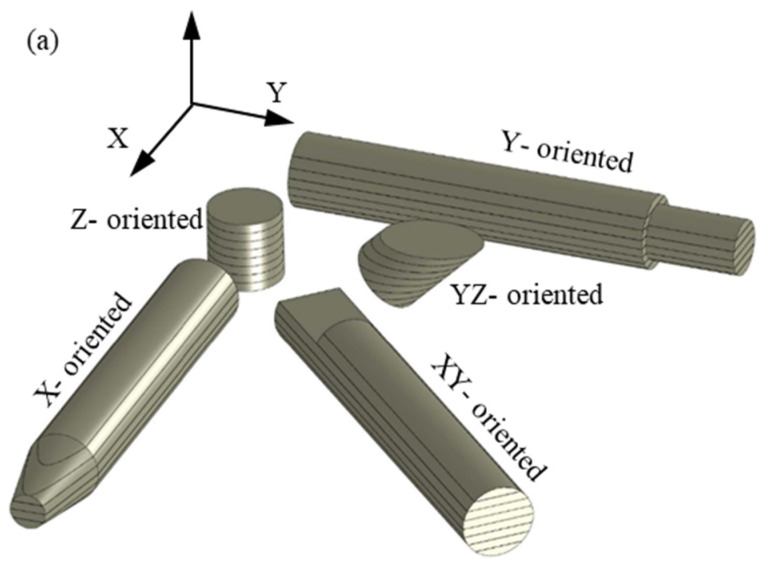

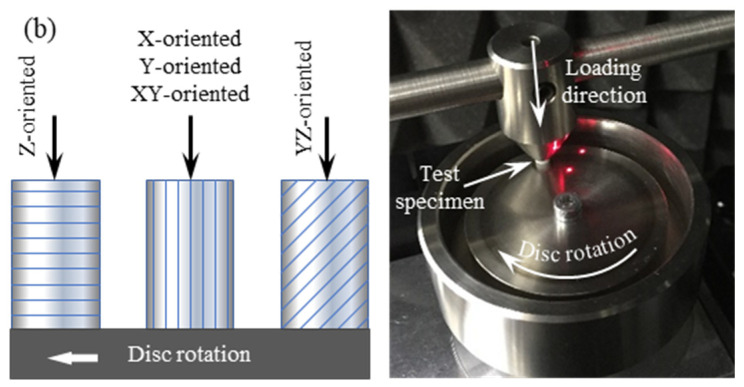
Layer orientations of test specimens (**a**) in the SLS build chamber and (**b**) during experiments.

The glass bead-filled PA12 test specimens, in the form of pins with a 4 mm diameter and a 25 mm length, were fabricated by the SLS technique in an EOS P380 system using EOS-PA 3200 GF tribological-grade powder. Fabrication parameters are given in Table 1. In the build chamber, the specimens were oriented along the X-axis, Y-axis, Z-axis, at 45° to the X-axis (XY-plane), and at 45° to the Z-axis (YZ-plane), as shown in Figure 1a. For easy sorting of the fabricated specimens, different micro-cuts were introduced in the CAD models to mark the different orientations and in such a way that it did not affect the load-carrying capacity of the specimen (see Figure 1b). The test specimens were built along the positive Z-axis, and a set of three test specimens was manufactured along each orientation. The selected build orientations were to yield three different layers’ orientations relative to the sliding plane of the stainless-steel disc (see Figure 2). The tribo-surfaces of the test specimens were the whole circular pin ends. Once the fabrication process was completed, the parts were taken off the support and cleaned with sandblasting to remove any sticking powder. Post-processed active tribo-surfaces were then kept out of dirt, humidity, and any oily substances.

### 2.2. Pin-on-Disc Wear Test

**Figure 3:**
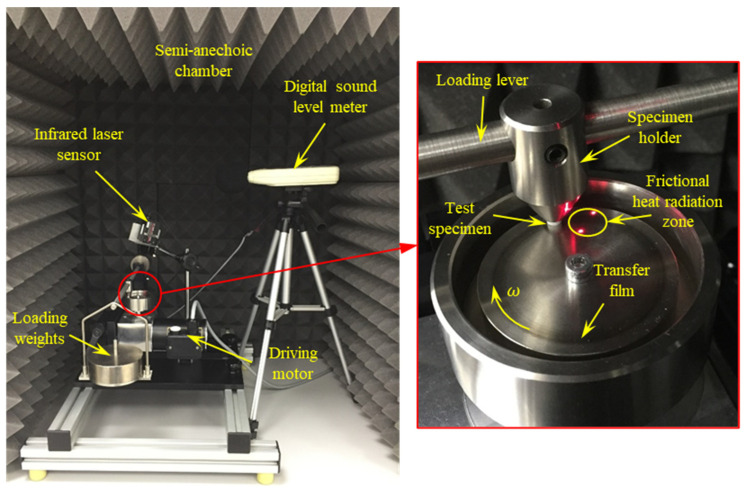
Experimental test rig with the pin-on-disc test apparatus.

In this work, the tribological properties of PA 3200 GF pin-shaped specimens were examined on a TM 260 pin-on-disc wear test apparatus (see Figure 3). The pin-shaped test specimen was inserted in the pin holder and loaded against the rotating stainless-steel disc. The mean radius of the sliding track was 20 mm, and its width was 4 mm (i.e., the specimen diameter). The normal load on the test specimen was applied by means of a dead weight hung at the lever end. The device was designed in such a way that the normal load (N) on the test specimen was twice the dead weight. The rotation speed of the steel disc was controlled by means of a drive unit through a gear reducer to produce a maximum sliding speed of 0.5 m/s, which corresponded to a rotation speed of 240 rpm. The experimental conditions are given in Table 2. Before running the experiments, measures were taken to ensure that the pin axis was normal to the disc surface, and the axial run-out of the disc was negligible (less than 0.02 mm). To ensure dry-sliding conditions, the disc surface was cleaned with 99.9% ethanol to remove any dirt, oily substances, or adsorbed matter; furthermore, the experiments were carried out in a low-humidity environment (less than 10%).

The frictional heating temperature of disc surface close to the contact zone was measured using an infrared laser sensor (KEYENCE-FT-H30), along with a digital amplifier unit FT-50AW. The infrared laser sensor detected the emitted frictional heat from a circular area with a 6 mm diameter around the sliding track after about 40 ms of disengagement.

**Figure 4:**
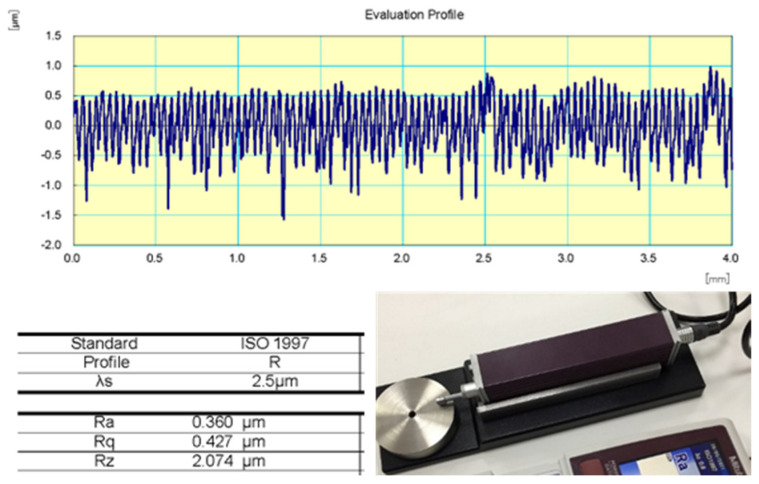
Roughness measurement of the sliding track (4 mm width).

As polymer friction results in the formation of a polymer transfer film on the countersurface, which in turn affects the performance of tribo-contacts, several stainless-steel discs of 50 mm diameter were manufactured with almost similar surface roughness, so a fresh disc was used with every test specimen. The surface roughness of all discs was measured using SURFTEST SJ-210, and the measured (Ra) roughness value normal to the sliding track (r = 18–22 mm) was around 0.35 ± 0.03 microns (see Figure 4). In the present experiments, one test specimen along each orientation was examined, since the polymers’ tribological characteristics are very sensitive to small variations of contact surfaces.

### 2.3. Noise Measurement

The friction-induced noise was measured using a digital sound level meter (GM1357), with 30–130 dB measurement range and an accuracy of ±1.5 dB. Measurements were carried out in a semi-anechoic chamber constructed as a wooden box lined with a high-density and echo-friendly polyurethane foam. The acoustic foam was capable of damping out low-to-mid noise frequencies inside the room. The recorded background noise, before running the experiments, was 35–37 dB. During the experiments, several measures were taken to exclude noise from external sources.

### 2.4. Data Acquisition

The pin-on-disc wear test apparatus was equipped with a double-bending beam force sensor (ME-KD60). The output signal of the KD60 load cell was fed into the NI-9237 module through NI 9949 (RJ-50 to a Screw Terminal Accessory). The module accessed the data at a rate of 50 kS/s and was connected to the NI-compactDAQ controller. A moving average function with Labview was used to smooth out the signal and record the friction force value at 500 ms intervals. On the other hand, an Arduino Uno microcontroller board was used to convert the 4–20 mA output of the infrared laser temperature sensor into 0–5 V signal, and a real-time averaging function was used to smooth out the sensor signal and record the temperature at 500 ms time intervals. Similarly, the DC output signal of the digital sound level meter of 10 mV/dB was handled with an Arduino Uno board, and the friction-induced noise level was recorded after smoothing at time intervals of 500 ms.

### 2.5. Tribo-Surface Characterization

For tribo-surface characterization, the worn surfaces of PA 3200 GF test specimens were coated with a 100 Å thin film of gold in a JFC-1100E ion sputtering device and then scanned with a JSM-5400LV scanning electron microscope (SEM).

## 3. Results and Discussions

Throughout the present experiments, the pin-shaped PA 3200 GF specimens were loaded against the rotating steel disc by means of a loading lever in such a way that the normal load on the pin was 50 N, and the disc was rotated at 120 rpm. To experimentally simulate more realistic operating conditions, the experiments were run for a duration of 45 min, which was sufficient enough to attain steady-state tribological properties of the composite material. As the diameter of the test specimen was 4 mm, the average contact pressure (P) was about 4 MPa. Indeed, the real contact pressure was much higher than the average value, since the real contact area was much smaller than the apparent area of contact; the real contact area was 10–30% of the apparent contact area [25]. Additionally, the radius of the sliding track on the steel disc was 20 mm, which maintained a sliding speed of 0.25 m/s. Consequently, with such levels of contact pressure and dry-sliding speed, the test specimens could be considered as operating under heavy loading conditions.

The effects of build orientation on the properties of fabricated objects were mainly attributed to two main factors: the delay time between scanning vectors (hatching pattern) and the layer orientations relative to the loading direction. Indeed, the wear depth in the current experiment was less than the layer thickness (150 μm), and hence, the effect of the former factor on the tribological properties of test specimens became inferior. Furthermore, current SLS systems evolved to the degree that the optimum hatching pattern was set automatically to maximize object density and reduce the porosity of fabricated objects. On the other hand, by excluding the effect of the hatching pattern, the layer orientations relative to the loading direction became the ruling factor that affected the tribological properties of PA 3200 GF-fabricated objects. Therefore, the discussions in the following sections will be focused on the effects of layer orientations relative to the sliding plane on the tribological properties of PA 3200 GF composites.

### 3.1. Coefficient of Friction (COF)

The dry friction at the polymer/metal interface was attributed mainly to two processes: (1) polymer surface deformation as a result of the ploughing and/or cutting action of hard surface asperities into the polymer surface and (2) the shearing of adhesive junctions formed at regions of real contact between the polymer and the hard surface [26]. In fact, the deformation component of friction is of lower effect in thermoplastics, and most frictional resistance is attributed to the adhesion between sliding surfaces; therefore, introducing lubrication reduces friction considerably, regardless of the countersurface roughness [27].

**Figure 5:**
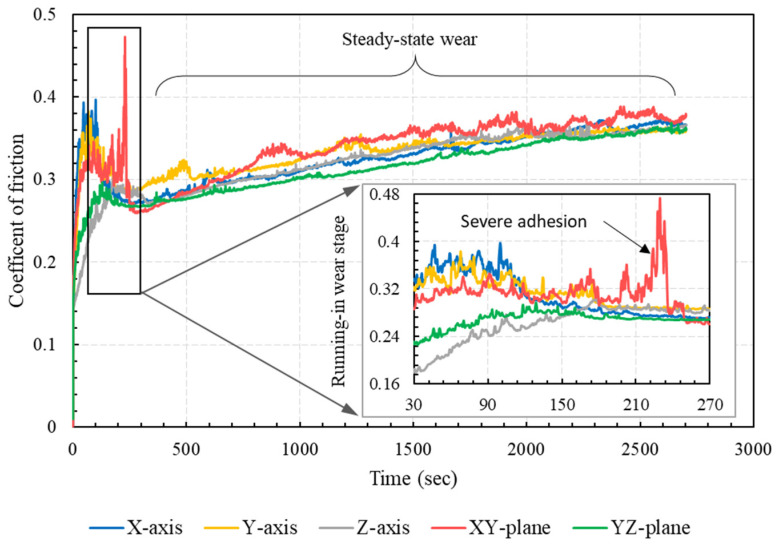
Coefficient of friction variation with time for different specimen orientations.

The COF of PA 3200 GF specimens sliding against steel was obtained by dividing the measured friction force by the applied normal load. The evolution of the COF with times for specimens with different build orientations is shown in Figure 5. The results showed that there was a sharp increase in the COF at the beginning of the experiment, which corresponded to the running-in stage (early 5 min of dry sliding). This sharp increase was owed mainly to the gradual increase in the real contact area, where more adhesive junctions were formed and sheared away, causing such a sharp increase in the coefficient of friction. Additionally, it was noticeable that the parts built along the Z-axis and the YZ-plane exhibited comparatively lower COF during the running-in stage, while the specimen built in the XY-plane had the highest COF during this stage. After the running-in stage, the results showed that all specimens, except those built along the XY-plane, had very similar values of COF. The specimen built along the XY-plane showed unstable frictional properties throughout the experiment duration.

**Figure 6:**
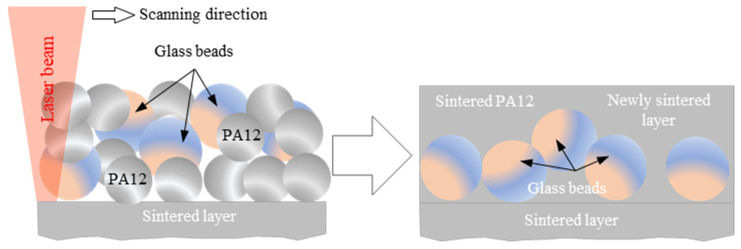
Possibility of glass bead precipitation in the sintered layer.

A possible explanation for the relatively low COF of specimens built along the Z-axis and the YZ-plane might be the sintering-induced inhomogeneity of glass bead distribution in the sintered layers. Since the glass beads (soda lime glass) had a higher density compared to PA12 (2.5 g/cm^3^ vs. 1.02 g/cm^3^), the glass beads tended to precipitate in the layer during the laser-sintering process, motivated by the low melt viscosity of PA12. (See Figure 6 for the schematic description of this possibility.) During experiments, more precipitated glass beads slid against the steel disc and were worn mainly by abrasion, which resulted in lower values of the COF. On the other hand, the unstable frictional properties of the XY-oriented specimen, manifested by the fluctuating COF, was attributed mainly to the severe adhesion at the polymer/steel interface [26].

### 3.2. Friction-Induced Noise

The friction-induced noise of polymer composites sliding against a hard surface was attributed mainly to the stick–slip phenomenon, which predominates at low sliding speeds (few mm/s) [28]. At higher sliding speeds, higher levels of friction noise are associated with the rapid formation and breakdown of adhesive junctions. However, this explanation does not rule out the contribution of abrasion to the induced noise.

**Figure 7:**
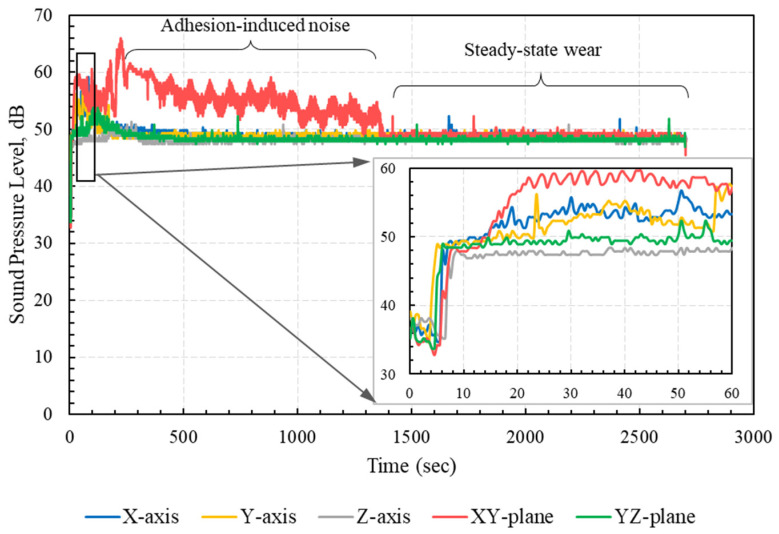
Friction-induced noise of test specimens with different orientations.

**Figure 8:**
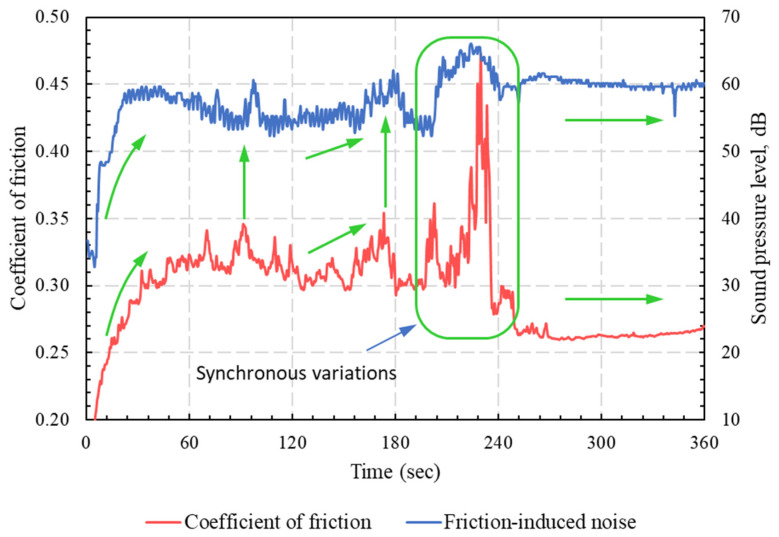
Synchronous behavior of the friction coefficient and the friction-induced noise for XY-oriented specimens.

The measured friction-induced noise levels of specimens with different build orientations are shown in Figure 7. The results showed clearly that higher noise levels were associated with higher COF. Accordingly, the specimens built along the Z-axis and the YZ-plane exhibited low friction-induced noise, whereas the specimen built along the XY-plane showed the highest level of frictional noise, corresponding to the highest COF levels. Moreover, at a steady-state region where the COFs of all specimens were very close, the friction-induced noise levels were very close. The synchronous variations of friction-induced noise and friction coefficient are further depicted in Figure 8. The figure shows synchronous trends of friction-induced noise and COF at the running-in stage of the specimen oriented along the XY-plane; the noise increased as the friction force increased and vice versa. These results are in good conformity with previous findings that higher COF values (larger than 0.3) are associated with strong interfacial adhesion [26]. In most cases, such strong adhesion, when combined with anisotropic cohesion in the polymer matrix, can result in the subsurface shearing of the polymer rather than shearing the interface. On the other hand, it has been reported that weak interfacial adhesion is associated with low COF (less than 0.1), and junction shearing occurs at the interface [26].

### 3.3. Frictional Heating

**Figure 9:**
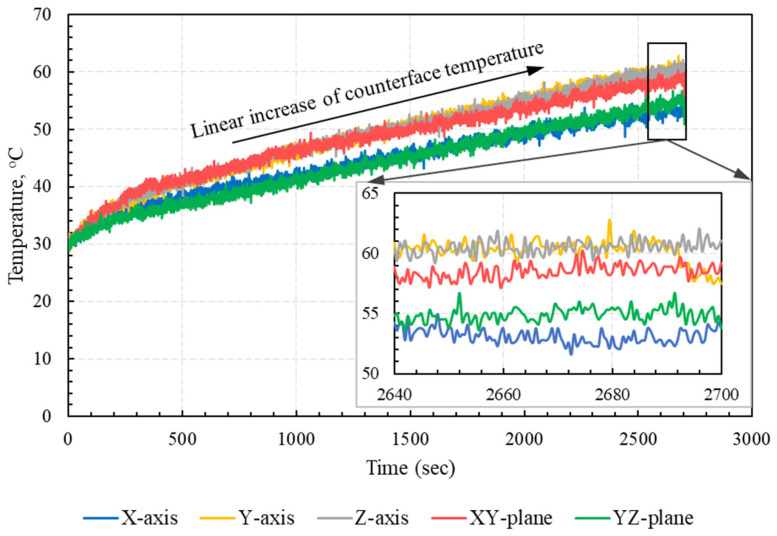
Approx. interface temperatures for different build orientations.

It was estimated that most of the work performed against friction is converted into heat energy at the sliding interface [27]. Additionally, surface deformation, including ploughing and/or cutting, is considered a major source of subsurface heating, while adhesion can induce surface, as well as subsurface, heating. Furthermore, repeated formation and shearing of adhesive junctions contributes to the pronounced vibrations of subsurface molecules, which result in subsurface heating [26,29]. As PA12 is a semi-crystalline polymer, sintered PA12 includes regions of amorphous structure, as well as regions of crystalline structure. Since the crystallinity percent of PA12 powder was about 49.1%, while the crystallinity of laser-sintered PA12 dropped to 24.5% [30], most of the contact area of PA 3200 GF was of an amorphous microstructure. Meanwhile, the stiffness of amorphous regions in laser-sintered PA12 fell rapidly as the interface temperature rose above the glass-transition temperature [27,29]. Thus, frictional heating significantly affected the tribological properties of polymers and polymer composites, as it could result in the pronounced softening of amorphous regions. Consequently, as the interface temperature increased, the adhesion component dominated the frictional behavior at the PA 3200 GF/steel interface. The disc temperature at the vicinity of PA 3200 GF/disc contact region was measured with an infrared laser sensor, and the measured temperature was used as a reasonable approximation of the interface temperature, since it was not straightforward to measure it directly. The approximate interface temperature measurements are plotted in Figure 9 for specimens with different build orientations. The results showed that the specimens built along the X-axis and in the YZ-plane experienced low interface temperature, compared with those built along other orientations. The results further emphasized the mutual effects of COF and interface temperature, where a higher COF resulted in high interface temperature, which, in turn, softened the amorphous regions in the substrate layer, causing strong adhesion and further increase in the COF.

The results clearly showed that the interface temperature gradually increased for all specimens. However, it is noteworthy that the measured temperature was affected by the reflectivity of the disc surface in the vicinity of the contact zone, which, in turn, was affected by the thickness and homogeneity of the transfer film. Additionally, as the pin slid over the disc, the real contact area increased, making room for a more adhesive junction to be formed and sheared away, which resulted in a gradual increase in the interface temperature. Therefore, both the change in disc surface reflectivity and the increase in real contact area contributed to the shown increase in the measured interface temperature.

### 3.4. Wear Rate (Wear Coefficient)

**Figure 10:**
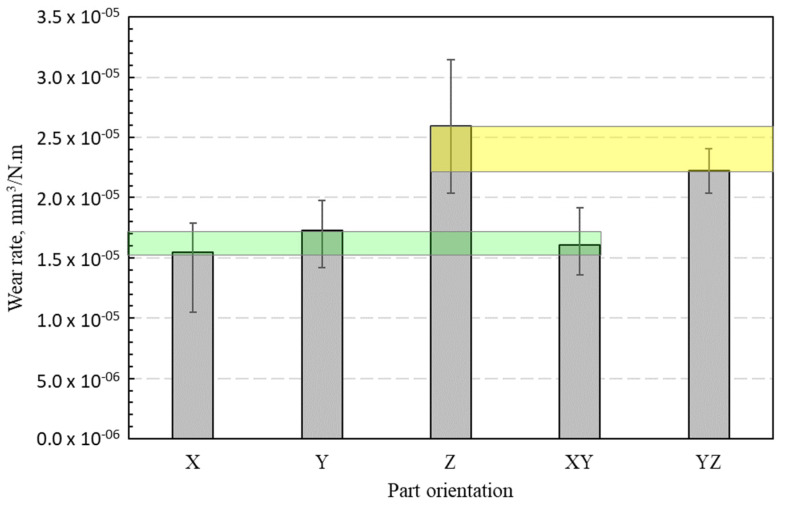
Wear rate of specimens with different build orientations (45 min).

The wear of specimens’ tribo-surfaces occurred as a result of the abovementioned modes of friction. The observed modes of wear in the dry sliding of PA 3200 GF against the steel disc were abrasive wear, adhesive wear, and surface fatigue wear. The specific wear rate (*K_s_*) in (mm^3^/N.m) was calculated as [31];

(1) $$K_{s}=\frac{\Delta V}{P\times L}$$

where ΔV is the wear volume (mm^3^), *P* is the applied load (N), and *L* is the sliding distance (m). Figure 10 shows the specific wear rates of PA 3200 GF specimens with different build orientations after 45 min of dry sliding against the steel disc. The figure shows clearly that the parts built along the X-axis, Y-axis, and XY-plane exhibited the lowest wear rate, while the specimens built along the Z-axis and the YZ-plane experienced a high wear rate. In other words, the PA 3200 GF specimens with their layers oriented normally to the steel disc (sliding surface) showed lower wear rates than those with layers oriented parallel or inclined to the disc surface. The results from this figure highlight the importance of choosing the appropriate layer orientation relative to the sliding plane for the purpose of controlling the wear rate of the PA 3200 GF composite.

The average values of the tribological parameters of PA 3200 GF sliding against the steel disc for a duration of 40 min (after 5 min of running-in stage) are shown in Table 3. The tabulated average values should be handled with caution, since they are valid only for the described experimental conditions and cannot be further extrapolated for other conditions.

### 3.5. Scanning Electron Microscopy (SEM)

**Figure 11:**
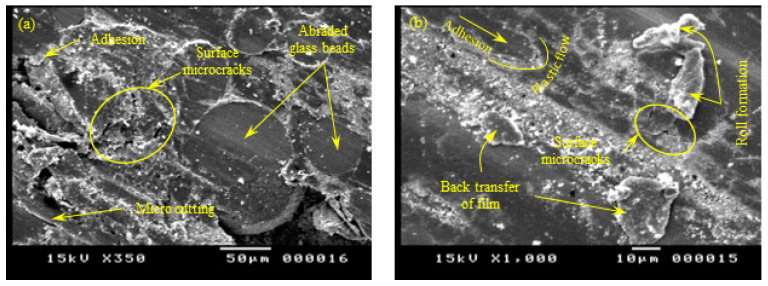
SEM of the tribo-surface for the X-oriented specimen (50 N, 0.25 m/s, and 45 min) at 350× (**a**) and 1000× (**b**) magnification.

**Figure 12:**
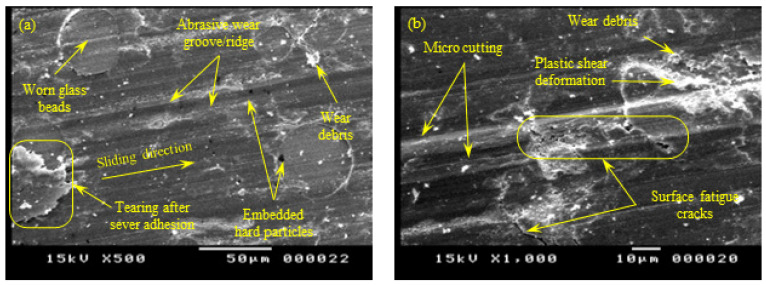
SEM of the tribo-surface for the Y-oriented specimen (50 N, 0.25 m/s, and 45 min) at 500× (**a**) and 1000× (**b**) magnification.

**Figure 13:**
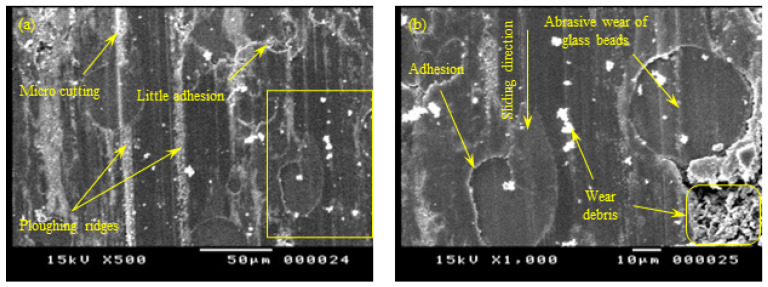
SEM of the tribo-surface for the Z-oriented specimen (50 N, 0.25 m/s, and 45 min) at 500× (**a**) and 1000× (**b**) magnification.

**Figure 14:**
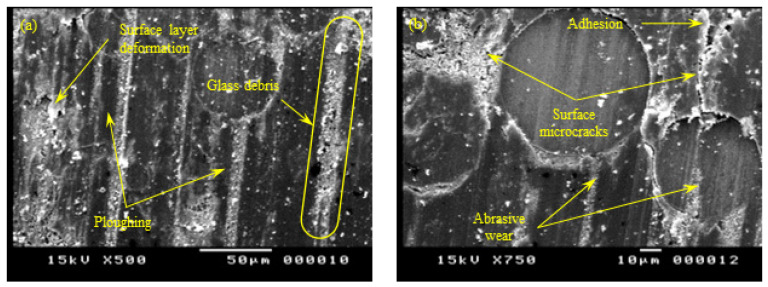
SEM of the tribo-surface for the YZ-plane specimen (50 N, 0.25 m/s, and 45 min) at 500× (**a**) and 750× (**b**) magnification.

**Figure 15:**
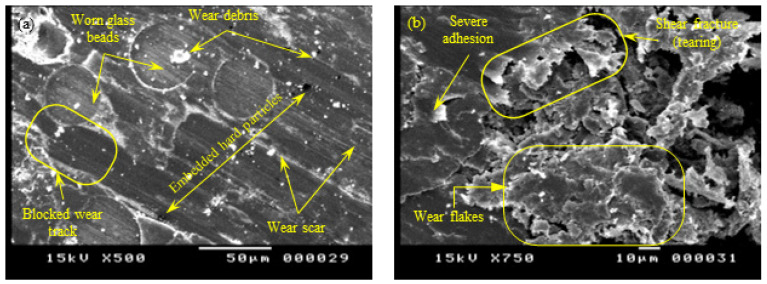
SEM of the tribo-surface for the XY-plane specimen (50 N, 0.25 m/s, and 45 min) at 500× (**a**) and 750× (**b**) magnification.

Characterization of the tribo-surface is an extremely important tool for controlling the friction and the wear behavior of polymer composites. Therefore, the sliding surfaces of PA 3200 GF specimens built along the five different orientations were investigated using a scanning electron microscope (SEM), and the results are shown in Figure 11, Figure 12, Figure 13, Figure 14 and Figure 15. The SEM of the tribo-surface of the X-axis oriented specimen, shown in Figure 11, revealed the following important remarks: (1) abrasive wear was the dominant wear pattern of glass beads, while adhesive wear, abrasive wear, and surface fatigue wear patterns coexisted side-by-side in regions of sintered PA12; (2) the existence of surface microcracks that were perpendicular to the sliding direction accentuated the prominent role of the adhesive wear pattern; (3) the roll formation, which is shown in Figure 11b, was the result of strong adhesion at real contact areas, which caused the initiation of surface and/or subsurface cracks. Repeated frictional traction on the surface resulted in crack propagation and later, coalescence, to form wear debris that rolled between the sliding surfaces and deformed in the shape of long rolls [32]; (4) the worn surface was characterized by the presence of wear flakes that might be the result of back transfer from the countersurface; and (5) worn glass beads resulted in the appearance of crushed glass powder on the sliding surface.

Figure 12 shows the SEM of the worn sliding surface of the Y-oriented specimen. The worn surface was characterized by the appearance of embedded hard particles in the PA 3200 GF sliding surface; this was a case of three-body abrasion, in which hard particles were peeled out of the steel disc surface and penetrated the PA 3200 GF surface. Later, the embedded steel particles acted as an emery cloth, which caused the abrasive wear of the disc surface [29].

In addition, Figure 12 shows pronounced abrasive wear in the form of micro-cutting marks at PA12 regions, as well as abrasive marks in glass beads. Moreover, the surface showed some areas with severe tearing as a result of strong adhesion with the disc surface that resulted in the localized heating and softening of the PA12 region, which, in turn, caused excessive deformation/elongation in this region. In some regions of PA 3200 GF, adhesion caused the appearance of surface fatigue cracks normal to the sliding direction. Furthermore, the tribo-surface in Figure 12 shows surface micro-cracks with random orientations, which may originate as a result of surface/subsurface voids in poorly sintered regions.

Figure 13 shows the SEM of the Z-oriented specimen. Unlike X-oriented and Y-oriented tribo-surfaces, abrasive wear predominated the tribo-surface of Z-oriented specimens. The sliding surface of Z-oriented specimens showed clear marks of ploughing in some regions and micro-cutting in others. The observed abrasive wear ruled both the PA12 and the glass bead regions. On the other hand, limited signs of adhesive wear were observed. The SEM micrograph of the Z-oriented tribo-surface further supported the hypothesis of glass bead precipitation in the sintered layer (see Figure 6). Moreover, the presence of tiny-sized wear debris of glass beads further supported the mentioned hypothesis.

Similarly, the SEM of the YZ-oriented tribo-surface, shown in Figure 14, suggests that abrasive wear predominated the wear pattern of the PA 3200 GF sliding surface. The sliding surface featured clear ploughing marks and several micro-cutting tracks through the PA12 and glass bead regions. Additionally, the type of existing wear debris was the type that commonly results from the abrasion of a brittle matter (i.e., glass beads). In contrast, limited signs of adhesive wear affirmed the conclusion that PA 3200 GF specimens with layers oriented parallel or inclined to the sliding surface were worn mainly by abrasion.

In contrast to the Z-oriented tribo-surface, the SEM of the XY-oriented sliding surface (see Figure 15) showed clear signs of severe adhesive wear, which was manifested by surface tearing and the appearance of elongated tongues. The results from Figure 15 are in excellent conformity with those shown in Figure 5, Figure 6, Figure 7, Figure 8 and Figure 9, which show that strong adhesion resulted in high COF, higher levels of friction-induced noise, and elevated interface temperature. The induced frictional heating further softened the surface layer of PA 3200 GF, motivating adhesion in more contact regions and thus maintaining a closed loop of tribological events. Another significant feature of the XY-oriented tribo-surface is the presence of accumulated wear flakes. Indeed, severe adhesion combined with anisotropic cohesion of the polymer surface layer caused tearing in the form of irregular-shaped wear flakes. Free wear flakes can either roll between sliding surfaces (i.e., roll formation) or transfer to the countersurface [33,34,35,36]. Hence, a transfer film of PA12 is formed on the disc surface, which may partially or fully cover the wear track. However, the cyclic thermal loading of the transfer film may cause some flakes to peel out and transfer back to the polymer surface. Therefore, the relatively high wear rate of the XY-oriented specimen suggests that the transfer film was peeled out more frequently; thus, the polymer continued to transfer to the disc surface. In effect, a stable transfer film can decrease the wear rate, since it was found that polymers are reluctant to transfer further onto their own transfer films [27].

## 4. Conclusions

The selective laser sintering (SLS) technique has evolved to the point that it is being used extensively in the manufacturing of functional parts and with moderate production capacity. However, the properties of laser-sintered parts are orientation-dependent. Therefore, the present study aimed to investigate the orientation-dependent tribological properties of glass bead-filled PA12 (PA 3200 GF) composite to facilitate the SLS fabrication of parts with customized tribological characteristics. The friction and wear properties of pin-shaped specimens with five different build orientations were examined on the pin-on-disc tribo-tester. Additionally, interface temperate and friction-induced noises were measured. Experiments were carried out under a normal load of 50 N, a sliding speed of 0.25 m/s, and for 45 min. The results suggest that the layers’ orientation relative to the sliding plane was a ruling parameter that determined the dominant wear pattern. Accordingly, where the build layers were parallel or inclined to the sliding plane, abrasive wear predominated and wear rate increased. By contrast, adhesive wear dominated when the build layers were perpendicular to the sliding plane, and wear rate decreased. In addition, the results elucidated a noticeable synchronous variation pattern of adhesion and friction-induced noise. Moreover, the SEM of the PA 3200 GF sliding surface suggests that where adhesion occurred and polymers transferred to the countersurface, transfer film could not adhere efficiently to the steel disc, and eventually, back-transfer occurred; the process continued, thereby causing an increase in the wear rate.

## Figures and Tables

**Table 1:** SLS of PA 3200 GF test specimens.

Powder	PA3200GF
SLS machine	EOS P380 Machine
Layer thickness (mm)	0.15
Outline power (W)	14.48 and 700
Outline scanning speed (mm/s)	700
Hatching power (W)	22.4
Hatching speed (mm/s)	2000
Scan spacing (mm)	0.3

**Table 2:** Experimental conditions on the in-on-disc wear test apparatus.

Normal load (N)	50
Rotation speed (rpm)	120
Sliding speed (m/s)	0.25
Sliding track radius (mm)	20
Test duration (min)	45
Initial temperature (°C)	29–30
Background noise level (dB)	35–37
Humidity (%)	7–10

**Table 3:** Average values of steady-state performance parameters.

Average Steady State	Specimen Orientation				
	X	Y	Z	XY	YZ
COF	0.3301	0.3369	0.3299	0.3435	0.3199
Noise level (dB)	48.9	48.8	48.1	51.6	48.3
Approx. interface temperature (°C)	45.5	50.4	50.4	49.8	45.2
Avg. wear depth (mm)	0.042	0.047	0.07	0.043	0.06
Wear rate (mm^3^/N.m)	1.54 × 10^−5^	1.73 × 10^−5^	2.59 × 10^−5^	1.61 × 10^−5^	2.22 × 10^−5^

## Data Availability

The data are contained within the article.
